# Elements of Return-to-Work Interventions for Workers on Long-Term Sick Leave: A Systematic Literature Review

**DOI:** 10.1007/s10926-024-10203-0

**Published:** 2024-06-07

**Authors:** Christa J. C. de Geus, Maaike A. Huysmans, H. Jolanda van Rijssen, Marianne de Maaker-Berkhof, Linda J. Schoonmade, Johannes R. Anema

**Affiliations:** 1 Department of Public and Occupational Health, Amsterdam Public Health Research Institute, Amsterdam UMC, Vrije Universiteit Amsterdam, Van Der Boechorststraat 7, 1081 BT Amsterdam, The Netherlands; 2 Dutch Institute of Employee Benefit Schemes (UWV), Amsterdam, The Netherlands; 3 Research Centre for Insurance Medicine, AMC-UMCG-VUmc-UWV, Amsterdam, The Netherlands; 4 University Library, Vrije Universiteit Amsterdam NL, De Boelelaan 1117, P.O. Box 7057, 1007 MB Amsterdam, The Netherlands

**Keywords:** Disability pension, Vocational rehabilitation, RTW, Work disability, Intervention

## Abstract

**Purpose:**

The aim of this systematic review is to identify vocational rehabilitation (VR) interventions that are effective to enhance return-to-work (RTW) for people on long-term sick leave (> 90 days) and to identify main elements of these interventions.

**Methods:**

Six electronic databases were searched for peer-reviewed studies published up to February 2022. Each article was screened independently by two different reviewers. Thereafter, one author performed the data-extraction which was checked by another author. The EPHPP quality assessment tool was used to appraise the methodological quality of the studies.

**Results:**

11.837 articles were identified. 21 articles were included in the review, which described 25 interventions. Results showed that ten interventions were more effective than usual care on RTW. Two interventions had mixed results. The effective interventions varied widely in content, but were often more extensive than usual care. Common elements of the effective interventions were: coaching, counseling and motivational interviewing, planning return to work, placing the worker in work or teaching practical skills and advising at the workplace. However, these elements were also common in interventions that were not effective on RTW compared to usual care and can therefore not explain why certain interventions are effective and others are not.

**Conclusion:**

The effective interventions included in this study were often quite extensive and aimed at multiple phases of the RTW-process of the worker. In the future, researchers need to describe the population and the content of the investigated interventions more elaborate to be able to better compare VR interventions and determine what elements make interventions effective.

**Supplementary Information:**

The online version contains supplementary material available at 10.1007/s10926-024-10203-0.

## Background

Long-term sickness absence has consequences for a person’s social and psychological well-being [1–3] and leads to high costs for society [4, 5]. Thus, it is important for these people to return to work sustainably as fast as possible. To increase their chances of returning to work, they are often supported in their return to work process, by offering them vocational rehabilitation (VR) interventions. Scientific evidence for the elements that these VR interventions should consist of to make them effective is lacking.

Literature shows that VR interventions are most effective if they are offered early in the return to work (RTW)-process [6] and thus prevent people from becoming long-term work disabled. These studies show that a focus on RTW, behavioral activation, and on psychoeducation [7] and a multidisciplinary approach [6] are elements of effective VR interventions for people with a shorter sick leave duration. However even though there are examples of interventions that are effective for people with a longer duration of sick leave [8], it remains to be seen if these contain the same elements. A reason why different interventions are effective in a later stage of the RTW-process could be that people on long-term sick leave often experience multiple problems that play an important role in prohibiting them from returning to work [8, 9] that take more time to address. On top of this, they often suffer from multiple disorders [10], experience multiple psychosocial problems [11] and multiple social disadvantages [12] in comparison to people with shorter sick leave. VR interventions specifically targeting people on long-term sick leave should address these problems, to increase work participation of this group.

Currently, there are no reviews available that explore intervention elements of VR interventions that are effective on RTW for people on long-term sick leave, however a few reviews investigated which type of interventions are effective on RTW. A review by Aasdahl and Fimland [8] showed that more complex interventions, such as a combination of an occupational intervention and a clinical intervention, are effective on RTW for people with a long-term illness. An overview of systematic reviews by Levack [13] to examine the effectiveness of vocational intervention for people with a chronic illness on returning and maintaining work, showed that supported employment is an effective intervention for people with chronic illness. For other VR interventions no final conclusion was reached, due to a lack of high-quality studies.

In order to better understand why certain VR interventions are effective to support people on long-term sick leave with their return to work, while others are not, it is important to identify elements of these interventions that might explain their effectiveness. This insight can be used to develop more effective VR interventions. The only review that investigated the elements of effective interventions for people on longer-term sick leave focused exclusively on mental disorders [1]. This study showed that interventions with contact to the workplace (e.g. refamiliarization with workplace) and multicomponent interventions are effective. It is of interest to see if these elements are also present in interventions that are effective for people on long-term sick leave because of a wide range of disorders.

This review aims to identify which VR interventions are effective for the RTW of people on long-term sick leave or receiving a work disability pension for more than 90 days, regardless of the type of disorder they have. Additionally, this review aims to identify the main elements of these effective VR interventions in comparison to the usual care.

## Methods

We performed a systematic literature review. We included studies that investigated the effect of VR interventions on RTW among long-term (> 90 days) sick-listed workers in accordance with the Preferred Reporting Items for Systematic Reviews and Meta-Analysis (PRISMA) statement ([14]; www.prisma-statement.org). A protocol of this review was registered at PROSPERO (CRD42022104283).

### Search Strategy

A comprehensive search was performed in the bibliographic databases PubMed, Embase.com, APA PsycInfo (via Ebsco), Cinahl (via Ebsco), Scopus and the Cochrane Library from inception to February 7th 2022, in collaboration with a medical librarian (LS). Search terms included controlled terms (MeSH in PubMed, Emtree in Embase, Thesaurus terms in PsycInfo and Cinahl Headings in Cinahl) as well as free text terms. The following terms were used (including synonyms and closely related words) as index terms or free-text words: ‘return to work’ and ‘disability insurance’ and ‘interventions’. We used four blocks in our search strategy (appendix 1) to search studies that fit our PICO. Block 1 (e.g. absenteeism) and 2 (e.g. disability insurance) described the population and outcome, block 3 described the type of intervention (e.g. vocational rehabilitation) and block 4 described the type of study (e.g. controlled clinical trial). Since we included all types of usual care as control condition we did not include terms referring to a certain type of control. A search filter was applied to limit results to randomized controlled trials. The search was performed without date restrictions. Duplicate articles were excluded by a medical information specialist (LS) using Endnote X20.0.1 (Clarivate^tm^), following the Amsterdam Efficient Deduplication (AED)-method [15, 16]. The references of the included articles and known systematic literature reviews [6–8, 17, 18] on the same topic were checked for additional relevant studies. The full search strategies for all databases can be found in the Supplementary Information (Appendix 1).

### Selection Process

Two reviewers (CdG and MdMB) independently screened all potentially relevant titles and abstracts for eligibility using Rayyan [19]. Studies were included if they met the following criteria: (i) Employees and former employees of working age (18–67 years) who were (partially) sick-listed or received a work disability pension for at least 90 days on average at baseline, and who were absent from work for either work-related or non-work-related reasons. Studies which did not mention the sick leave duration of the participants or which included veterans were excluded; (ii) Interventions aimed at enhancing return to work or vocational rehabilitation. Medical interventions aimed at the treatment of the disease (e.g. drugs, surgeries) were excluded; (iii) Randomized controlled trial; (iv) Study involving RTW-related outcome measures (e.g. return-to-work, work retention, absenteeism, work status, competitive employment, time to RTW). Studies that were not published in a peer-reviewed academic journal or written in a different language then English were excluded. After screening based on title and abstract, the full text of the articles were independently checked by the two reviewers for the eligibility criteria. In case of disagreement, the disagreement was solved through discussion. If there was no consensus after discussion, a third reviewer (MH) decided if the article should be included in the review.

### Data Extraction and Synthesis

One author (CdG) extracted the data from the studies. This data was checked by another reviewer (MdMB). In case of discrepancies in the data extraction, the data extraction was discussed until consensus was reached. Data extraction to identify the different elements of the interventions was conducted by reviewer CdG and reviewer JvR, independently. Differences between the reviewers was discussed with reviewer MH.

Due to the expected heterogeneity of the included interventions a narrative synthesis was conducted based on the steps developed by Popay et al. [20]: 1) developing a preliminary synthesis, 2) exploring relationships in the data and 3) assessing the robustness of the synthesis product. In order to develop a preliminary synthesis and explore relationships in the data, we extracted data from the articles on general study elements (e.g. authors, year of publication, study design, type of outcome measure) and participant elements (number of participants, age, gender, type of disease, average sick leave duration, job elements). Next, the elements of the interventions were extracted from the included articles using a deductive synthesis. Based on the elements of the first six analyzed articles and categories used in three other reviews [6, 7, 21] a framework was developed. The elements of the other articles were categorized according to this framework. If elements did not fit in the framework, new categories were added. The intervention elements according to the framework are described in Table 1.

**Table 1:** Description of intervention elements

Intervention elements	Description
**1. Preparing the worker for RTW**	**Aim of the intervention is to prepare the worker to RTW; at the end of the intervention, the worker is ready to find and start work**
Identifying problems for RTW	- Identifying barriers for RTW (either medical, psychological or social) - Identifying personal situation (e.g. work history, social situation) - Identifying functional status
Formulating rehabilitation plan	Describing the vocational rehabilitation process of the worker
Psycho-education aimed at RTW	Learning the worker how to deal with their pain or psychological problems, or improve their coping
Counselling/coaching/motivational interviewing	Aimed at stimulating and motivating the worker on different aspects of RTW
Cognitive behavioral therapy (CBT)/Acceptance and Commitment Therapy (ACT)/cognitive restructuring	Psychological interventions based on (aspects of) CBT, ACT or cognitive restructuring. Aimed at a cognitive or behavioral change. Can be aimed at different aspects such as goal setting, stress management, pain management
Physical training (outside the workplace)	Physical training, such as a functional restoration program, relaxation exercises or exercises aimed at increasing strength. Often aimed at reducing pain and increasing physical skills outside of the workplace
Targeting external factors	Targeting external factors, such as the financial situation and housing. Sometimes third parties are contacted to solve these problems
**2. Helping the worker find work**	**Aim of the intervention is to help the worker find work, train the worker in finding work or place the worker in work**
Teaching practical skills	Work-related training (e.g. training how to apply for a job)
Planning return to work	Formulating a plan on how to return to work
Searching for work	Searching work for the worker, contact with possible employers or giving worker advice on how to find work
Placing in work	Worker is placed in (paid) work
**3. Helping the worker remain in work**	**Aim of the intervention is to help the worker who has returned to work stay in work by offering advice, exercises or remaining in contact**
Physical training	Physical exercises at the workplace
Advice at work	Aimed at staying in work (e.g. workplace adaptions, contact with employer)
Follow-up meeting(s)	One or more booster sessions after the end of the intervention

Bold text is use to improve the readability of the table

We described the strength of our evidence based on the quality and quantity of the primary studies included in the review. Two reviewers (CdG and MdMB) independently evaluated the methodological quality of the full text papers using the EPHPP quality assessment [22]. The EPHPP tool was used to assess the quality of a study on six categories (selection bias, study design, confounders, blinding, data collection methods and withdrawals and drop-outs). For each category the studies were scored weak, moderate or strong. Based on these ratings a global rating was calculated. Studies received a strong global rating if they had no categories with a weak rating, a moderate global rating if they had one category with a weak rating or a weak global rating if they had two or more categories with a weak rating. The construct and content validity of the EPHPP tool have been demonstrated [23]. Differences in judgement were resolved through a consensus procedure. If consensus could not be reached, the item was discussed with reviewer MH and resolved.

## Results

### Study Search and Inclusion

**Fig. 1:**
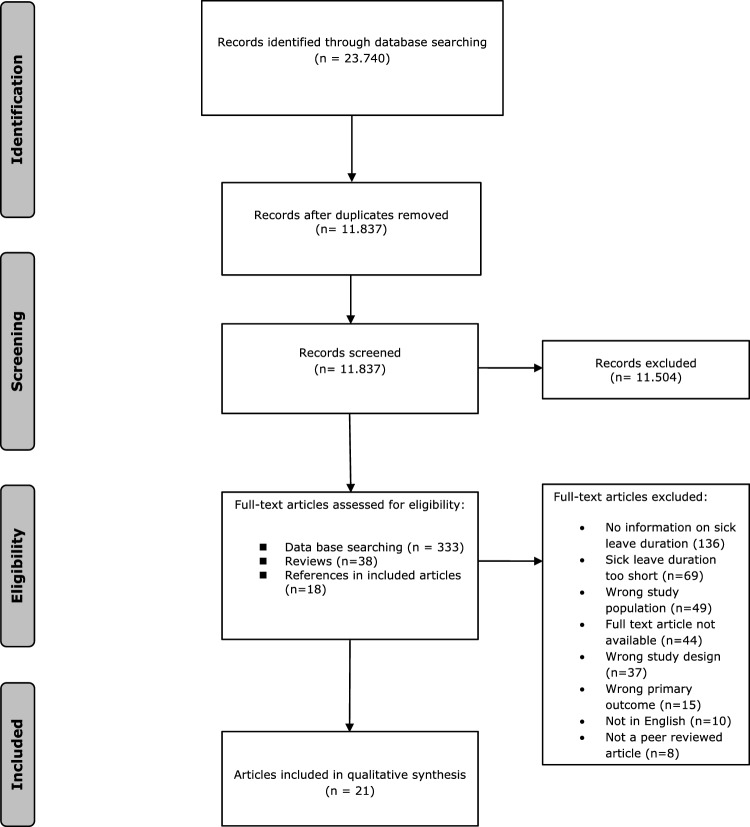
Flowchart of the search and selection procedure of studies

The literature search generated a total of 23,740 references: 4230 in PubMed, 3940 in Embase, 2211 in Cinahl, 975 in PsycInfo, 4886 in the Cochrane Library and 7498 in Scopus. The flow chart of the search and selection process is presented in Fig. 1. We identified 333 possible relevant articles in the database of which the full-text was assessed. During the search we identified 38 relevant systematic reviews of which the reference lists were assessed for eligible studies. At the end we also checked the reference lists of the eligible articles to identify additional articles. In total we checked the full text of 389 studies. Twenty-one studies were included. The 21 studies describe 25 different interventions. For one study a subsample of workers on long-term sick leave was used [24]. For another study the sample was divided in two subsamples based on sick leave duration (average sick leave duration of 3 months and 26 months) [25], we describe the outcomes of the intervention for both subsamples.

### Quality of Included Studies

Quality ratings are shown in Table 2. Of the 21 identified articles, eight were assessed as weak in the global rating [26, 28–33, 45], nine studies were assessed as moderate [24, 27, 34–40], and four studies were assessed as having a strong quality [25, 41, 42, 44]. Selection bias was present in many studies due to a low participation rate of the invited participants. Since only randomized controlled trials (RCT’s) were included, the study design was mostly assessed as strong. However, if the randomization was not properly described, study design was rated ‘moderate’. Blinding was often assessed as weak due to participants not being blinded to the condition (intervention or usual care group) they were allocated. The data collection methods were often assessed as weak, because self-reported questionnaires were used to measure return-to-work instead of more reliable methods, such as using data obtained from registries of social security institutes.

**Table 2:** Results of quality assessment (according to the criteria of the EPHPP quality assessment)

Article ID	Selection bias	Study design	Confounders	Blinding	Data collection methods	Withdrawals and dropouts	Global rating
Berglund [26]	weak	moderate	strong	moderate	weak	moderate	weak
Blonk [34]	weak	moderate	strong	moderate	strong	moderate	moderate
Cheng [27]	moderate	moderate	strong	moderate	weak	strong	moderate
Corey [28]	moderate	strong	strong	moderate	weak	weak	weak
Della-Posta & Drumond [35]	moderate	moderate	moderate	moderate	weak	strong	moderate
Drake [45]	weak	strong	weak	moderate	weak	strong	weak
Fleten [24]	moderate	strong	strong	moderate	strong	weak	moderate
Hees [29]	weak	strong	strong	moderate	weak	moderate	weak
Heinrich [30]	weak	strong	strong	weak	strong	strong	weak
Hellstrom [44]	strong	strong	strong	moderate	strong	moderate	strong
Huibers [31]	weak	strong	strong	moderate	weak	strong	weak
Lambeek [41]	moderate	strong	strong	moderate	strong	strong	strong
Li-Tsang [42]	moderate	moderate	strong	moderate	strong	strong	strong
Lytsy [36]	weak	strong	strong	moderate	strong	strong	moderate
Magnussen [37]	weak	strong	strong	moderate	strong	strong	moderate
Marhold [25]	moderate	moderate	strong	moderate	strong	strong	strong
Myhre [38]	weak	strong	strong	moderate	strong	strong	moderate
Nilsson [32]	moderate	moderate	strong	moderate	weak	weak	weak
Park[39]	strong	strong	strong	moderate	weak	strong	moderate
Van der Feltz [40]	weak	strong	strong	moderate	strong	moderate	moderate
Van Egmond [33]	weak	strong	strong	weak	strong	moderate	weak

### General Study Characteristics

General characteristics of the studies are presented in Table 3. Almost all studies had two arms (intervention and control) except for four studies with three arms [26, 30, 34, 36]. The number of participants ranged from 38 [32] to 2238 [45]. The mean sick leave duration ranged from 3 months [25] to 12.3 years [31]. Most studies (*n* = 12) included more women than men. Two studies [25, 36] only included women, all other studies included both men and women. The mean age ranged from 32.3 years [27] to 49.1 years [37]. Studies were mostly executed in Northern Europe (*n* = 16). Most common RTW outcome measures were RTW status, duration until RTW and claim duration. Only studies involving populations on sick-leave with a mean or median duration of more than 90 days were included, but within these studies the length of sick-leave varied considerably. Twenty-two interventions could be categorized based on the average or median sick leave duration of their sample or inclusion criterion. Nine studies concerned people with a sick leave duration with a median/mean of more than 3 months and less than one year. Twelve studies had a population on sick leave for 1 year or more (range: 12 months to 12.9 years). One study included both groups. Follow-up duration of the studies varied from “at program discharge” to 36 months. Eighteen interventions followed participants for at least 12 months (range 12 months-36 months).

**Table 3:** General study characteristics

Author, publication year, country	Sample; *N, sex* (% female), mean age (SD), (average) sick leave duration	Target population (average) sick leave duration	Intervention 1 General description Providers	Intervention 2 General description Providers	Comparison	Primary outcome measure Follow-up
Berglund et al. 2018, Sweden	**N** = 427 **Sex**: 93.9% female **Mean age**: 48.7 yrs (SD 8.3) **Average sick leave duration**: 7.7 years	People on long-term sick leave or temporary disability pension due to a mental illness and/or pain-related diagnosis	**Description**: Multidisciplinary Treatment aimed at identifying strengths and limitations for RTW and establishing an individualized rehabilitation plan which was followed **Providers**: Psychologist, physician, occupational therapist, social worker	**Description**: Acceptance and Commitment therapy. Psychological therapy to increase function and quality of life to increase psychological flexibility. Sessions were one hour long at the clinic or at home **Providers**: Psychologist	Usual care: regular contact with the SSIA/ SPES and healthcare providers	**Primary outcome measure**: RTW: proportion of wage due to more work increased **Follow-up**: At 12 months
Blonk et al. 2007, The Netherlands	**N** = 122 **Sex**: 19% female **Mean age**: 42 yrs (SD 7.9) **Average sick leave duration**: Not mentioned	Self-employed people with psychological complaints receiving a disability pension for more than 52 weeks	**Description**: Cognitive Behavioral Treatment (CBT): aimed at cognitive restructuring and registration of symptoms and situation. 11 two-weekly sessions of 45 min per session **Provider**: Psychologist	**Description**: Combined intervention: aimed at stress-management, advice on work processes and reducing workload, job demands and increasing decision latitude. 5/6 sessions of 1 h, twice a week **Providers**: Labour experts	No treatment: two brief sessions with general practitioner	**Primary outcome measure**: RTW: length of time until (partial) return to work **Maximum duration of observation period:** 360 days
Cheng & Hung 2007, Hong Kong	**N** = 103 **Sex**: 23.4% female **Mean age**: 32.3 yrs **Average sick leave duration**: 137.9 days (workdays lost since injury)	People with work-related rotator cuff tendinitis, with at least 90 days since claim filing/date of injury and physically fit to start functional training and work trial. Willing to participate	**Description**: Workplace-based work hardening training: aimed at improving physical health And improve functional restoration and work specific adaption **Provider**: Job coach	Not applicable	Usual care: clinic-based work hardening training	**Primary outcome measure**: RTW: resumption of occupational activities and type of duties **Follow-up**: At 4 weeks
Corey et al. 1996, Canada	**N** = 214 **Sex**: 32% female **Mean age**: 64% < 45 years; 36% > 45 years **Average sick leave duration**: 4.6 months (duration of disability)	People with a work-related soft tissue injury with disability longer than would be expected, totally disabled from work, receiving wage loss benefits at least 90 days after injury	**Description**: Functional restoration program: aimed at restoring function to lead to control, diminution and in some cases resolution of the pain **Provider**: Not mentioned	Not applicable	Usual care: referred back to treating physician with recommendations for proactive management	**Primary outcome measure**: RTW: work status **Follow-up**: Ranging from 9–27 months
Della-Posta & Drummond 2006, Australia	**N** = 39 **Sex**: 28.2% female **Mean age**: 41 yrs **Average sick leave duration**: 39.4 weeks	People who received deployment assistance, ready to secure employment and were looking for a new employer	**Description**: CBT + usual care: aimed at reducing depression, anxiety and stress levels and thus increasing perceived work capacity and work outcomes **Provider**: Not mentioned	Not applicable	CAU: job search program. Aimed at goal setting, resume preparation and job search skills	**Primary outcome measure**: RTW: Length of time until obtaining employment **Maximum duration of observation period:** 10 weeks
Drake et al. 2013, USA	**N** = 2238 **Sex**: 52.7% female **Mean age**: 43.5 yrs **Average sick leave duration**: 97.9 months on SSDI	People with schizophrenia or a mood disorder, interested in gaining employment who received a disability pension	**Description**: The intervention, based on the chronic care model (13) existing out of the individual placement and support model of supported employment, systematic medication management, and other behavioral health services **Provider**: Nurse, nurse care coordinator, team psychiatrist	Not applicable	Usual care: e.g. outpatient physician visits, medications and hospital care	**Primary outcome measure**: Employment status (any paid employment and competitive employment **Maximum duration of observation period:** 25 months
Fleten & Johnsen 2006, Norway	**N** = 332 **Sex**: 60.7% female (in entire population) **Mean age**: 40.4 years (in entire population) **Average sick leave duration**: 231.1 days	People with musculoskeletal or mental disorders who were sick-listed > 14 days	**Description**: Minimal intervention package: aimed at informing on available work measures, asking questions on the expected length of the current sick leave and on any relevant work adjustments for the ongoing sick leave **Provider**: Not applicable	Not applicable	Usual care: no information package	**Primary outcome measure**: Length of sick leave **Maximum duration of observation period:** 55 weeks
Hees et al. 2013, The Netherlands	**N** = 117 **Sex**: 53% female **Mean age**: 43 years **Average sick leave duration**: median: 4.8 months	People with a major depressive disorder who have a possibility to RTW and a relationship between the depressive disorder and the work situation and have At least 35% absenteeism and at least 8 weeks absenteeism or duration of depressive disorder > 3 months	**Description**: Occupational Therapy + TAU: aimed at problem clarification an intervention based on the Quality of Work model to create a balance between work demand and work capacity and a follow-up **Providers**: Occupational therapists	Not applicable	Treatment as usual: treatment by psychiatric residents/senior psychiatrist. Including psychoeducation, supportive therapy and cognitive behavioural interventions	**Primary outcome measure**: RTW: time until partial/full RTW and Absenteeism **Follow-up**: 6, 12, 18 months
Heinrich et al. 2009, The Netherlands	**N** = 254 **Sex**: 7.9% female **Mean age**: 45.2 years **Average sick leave duration**: 8.8 weeks (after 52 weeks waiting time)	Self-employed people with musculoskeletal disorder receiving a disability pension for more than 52 weeks	**Description**: PT: Physical Training without a cognitive behavioural component and workplace specific exercises aimed at improving physical capacity, to learn to cope with complaints and to stimulate correct postures/movements **Providers**: Physiotherapists or trainers	**Description**: PTCBWE: Physical Training with a cognitive behavioural component and Workplace specific Exercises: aimed at aim s to detect dysfunctional thinking habits and to change those thinking habits into a more realistic or functional way of thinking (in addition to the aims of the PT) **Providers**: Not mentioned	usual care: usual guidance by their general practitioner according to the guidelines of the Dutch College of General Practice for musculoskeletal disorders	**Primary outcome measure**: Claim duration (in days) the number of days the participant received work disability compensation between **Follow-up**: 6 & 12 months
Hellstrom et al. 2017, Denmark	**N** = 326 **Sex**: 67.8% female **Mean age**: 35 years **Average sick leave duration**: not mentioned	People with a mood and anxiety disorder and not ready to return to work within 3 months after inclusion	**Description**: IPS modified for people with recently diagnosed mood or anxiety disorder: aimed at supporting people to obtain and sustain competitive employment through mentor support **Providers**: Mentors and career counselors	Not applicable	Usual care: offered by job centers (e.g. mentor support)	**Primary outcome measure**: RTW: competitive employment or education at 24 months/ weeks until return to work **Follow-up**: 12 & 24 months
Huibers et al. 2004, The Netherlands	**N** = 151 **Sex**: 55% female **Mean age**: 43.5 years **Average sick leave duration**: 12.3 years	People with severe fatigue and on with complete absenteeism from work for 6–26 weeks	**Description**: CBT: aimed at diminishing fatigue, establish work resumption and other personal goals and to establish self-perceived recovery **Providers**: General Practitioners	Not applicable	No treatment: free to visit GP for usual care	**Primary outcome measure**: Absenteeism: self-reported work resumption or absenteeism (yes/no) at each measurement, and absenteeism registered by the occupational health service (number of partial or complete sick days in 365 days) **Follow-up**: 4,8, 12 months
Lambeek et al. 2010, The Netherlands	**N** = 134 **Sex**: 41.8% female **Mean age**: 46.2 years **Average sick leave duration**: not mentioned	People with low back pain for more than 12 weeks and were (partially) absent from work	**Description**: Integrated care: aimed at restore occupational functioning and achieve lasting return to work **Providers**: Clinical occupational physician, medical specialist, occupational therapist, physiotherapist	Not applicable	Usual care from their medical specialist, occupational physician, general practitioner, and/or allied health professionals	**Primary outcome measure**: RTW; duration of sick leave until full return to work **Maximum duration of observation period:** 12 months
Li-Tsang et al. 2008, China	**N** = 63 **Sex**: 38.1% female **Mean age**: 42.6 years **Average sick leave duration**: not mentioned	People with musculoskeletal injuries due to work with sick leave for over 7 months and have receive a training on work readiness program	**Description**: Job placement and support aimed at: of individual interviews, vocational counseling, job preparation and job seeking **Providers**: Case managers	Not applicable	Usual care: self-placement group: referred to workers health center helping them to search jobs	**Primary outcome measure**: RTW; working continuously for 4 weeks for > 18 h per week **Follow-up**: 3 weeks
Lytsy et al. 2017, Sweden	**N** = 308 **Sex**: 100% female **Mean age**: 48.5 years (SD 6.3) **Average sick leave duration**: 7.5 years (SD 3.2)	Women with mental illness and/or pain syndromes who were on sick leave or time-restricted disability pension	**Description**: Multidisciplinary assessments and individual rehabilitation: aimed at assessing symptoms, disability and functioning from different perspectives **Providers**: Physician, psychologist, occupational therapist and social worker	**Description**: ACT: form of CBT that uses acceptance and mindfulness strategies with behavioural strategies to increase function and quality of life **Providers**: psychologists	Usual care: provided by their regular health contacts	**Primary outcome measure**: RTW: measured with four different measures: returning to health insurance, number of reimbursed health insurance days, self-reported change in working hours, self-reported change in degree of engagement **Follow-up**: 12 months
Magnussen et al. 2007, Norway	**N** = 89 **Sex**: 65% female **Mean age**: 49 years **Average sick leave duration**: 10.69 years	People with back pain who receive full disability pension for more than one year	**Description**: Brief vocational-oriented intervention: aimed at lectures on spinal problems, motivational interviewing aimed at helping to focus on strength and capacity **Providers**: Counsellors from SSI or work office, physician, nurse	Not applicable	Control group: no further information	**Primary outcome measure**: RTW: being in a process of return to work defined as being on educational course or being in work training **Follow-up**: 12 months
Marhold et al. 2001, Sweden (1 – short term)	**N** = 36 **Sex**: 100% female **Mean age**: whole population: 46 years (SD 9) **Average sick leave duration**: 3 months	Women with neck-and shoulder pain or lower back pain on short-term (2–6 months) sick leave	**Description**: Cognitive behavioral return-to-work program aimed at focusing on skills needed to cope with pain and RTW **Provider**: Clinical psychologist	Not applicable	Treatment as usual: no cognitive-behavioral interventions	**Primary outcome measure**: Number of days on sick leave **Follow-up**: 6 months
Marhold et al. 2001, Sweden (2 – long term)	**N** = 36 **Sex**: 100% female **Mean age**: whole population: 46 years (SD 9) **Average sick leave duration**: 26 months	Women with neck-and shoulder pain or lower back pain on long-term (> 12 months) sick leave	**Description**: Cognitive behavioral return-to-work program aimed at focusing on skills needed to cope with pain and RTW **Provider**: Clinical psychologist	Not applicable	Treatment as usual: no cognitive-behavioral interventions	**Primary outcome measure**: Number of days on sick leave **Follow-up**: 6 months
Myrhe et al. 2014, Norway	**N** = 405 **Sex**: 46.4% female **Mean age**: 40.6 years **Average sick leave duration**: median 112 days	People with neck and back pain who were sick-listed between 4 weeks and 12 months and referred to an outpatient clinic	**Description**: Work-focused intervention + usual care: usual care and additional focus on RTW process **Providers**: physician, caseworker, physiotherapist	Not applicable	Control intervention: either comprehensive multidisciplinary intervention or brief multidisciplinary intervention aimed at removing fear-avoidance beliefs, restoring activity level, and enhancing self-care and coping	**Primary outcome measure**: RTW: first 5-week period to not receive a sickness benefits, a work assessment allowance pension, or a disability pension **Maximum duration of observation period:** 12 months
Nilsson & von Buxhoeveden 1996, Sweden	**N** = 38 **Sex**: 68.4% female **Mean age**: 40.5 years **Average sick leave duration**: median 290 days	People with work-related locomotor symptoms on long-term sick leave for at least 6 weeks—2 years	**Description**: Rehabilitation program existing out of occupational therapy and social counselling aimed at the pattern of sick leave absence and well-being versus pain and tension + CAU **Providers**: occupational therapist, social worker, physiotherapist, a psychiatrist or other physicians	Not applicable	No treatment program	**Primary outcome measure**: RTW: back in work or training/ rate of RTW **Maximum duration of observation period:** 36 months
Park et al. 2018, Canada	**N** = 728 **Sex**: 36.8% female **Mean age**: 45 years (SD 12.2) **Average sick leave duration**: 233.7 (SD 688) days (disability duration)	People (non) job attached with musculoskeletal disorder injured at work	**Description**: Motivational interviewing in addition to a standard functional restoration program aimed at strengthening the clients own motivation for change **Providers**: Motivational interviewing practitioner	Not applicable	Usual care: functional restoration program	**Primary outcome measure**: RTW: confirmed RTW status **Follow-up**: At time of program discharge
van der Feltz-Cornelis et al. 2010, The Netherlands	**N** = 60 **Sex**: 58% female **Mean age**: 42 years **Average sick leave duration**: 144 days	People with depressive, anxiety or somatoform disorders with at least six weeks of absenteeism	**Description:** Psychiatric consultation aimed at delivering a diagnosis and treatment plan, including suggestions for RTW adapted to the specific needs of the patients due to their specific disorder. OP's were trained in doing this **Providers**: Occupational physicians, psychiatrists and general practictioners	Not applicable	Care as usual: delivered by OP	**Primary outcome measure**: Time to RTW: Time to (lasting) RTW is defined as the period between the onset of sickness leave due to the mental disorder at hand and full RTW, for at least four weeks **Follow-up**: 3 & 6 months
van Egmond et al. 2016, The Netherlands	**N** = 171 **Sex**: 69% female **Mean age**: 48.4 years **Average sick leave duration**: not mentioned	People with cancer, sick-listed, receiving sickness or disability benefits, without employment on sick leave for at least 12 months and maximum 36 months	**Description:** The tailored RTW program: existing out of an introductory interview, a ‘Preparation for RTW’ part, and a ‘RTW’ part. Routes are tailored to the needs of the individual. + CAU **Providers:** re-integration coach, vocational therapists or personnel with background in HR,	Not applicable	CAU: The SSA’s usual care generally consisted of a few meetings per year with an insurance physician, and potentially also a labor market or re-integration expert	**Primary outcome measure:** Duration until sustainable (> 28 days) RTW **Maximum duration of observation period:** 12 months

Bold text is use to improve the readability of the table

### Study Results

Of the 25 different interventions, ten interventions were effective in improving the RTW outcome compared to usual care (Table 3). Two interventions showed mixed results; one intervention [40] showed that compared to usual care the intervention (advice from the psychiatrist to the occupational physician about RTW) was only effective on return to work rates at three months (58% versus 44%), while at six months this difference had disappeared (~ 85% in both groups). Another intervention, a cognitive behavioural RTW-program [25] showed only to be effective for people with relatively short-term sick leave (mean 3 months) but not for people with a long-term sick leave (mean 26 months). In addition, there was one study which reported a positive effect in favor of the usual care group at 6 months, compared to the group receiving physical therapy, but this effect was not present at 12 months [30].

### Elements of Interventions

Table 4 shows the characteristics and the elements of the interventions that were studied. Both effective and not effective studies varied widely in type of intervention. In general, studies examined the effect of psychosocial interventions, such as Cognitive Behavioral Therapy (CBT) [25, 30, 31, 34] and Acceptance and Commitment Therapy (ACT) [26, 36]. There were no differences in type of intervention when comparing the effective to the not effective interventions. Most (*n* = 15) of the interventions of which the duration was described (*n* = 20), lasted between 1 and 6 months. Six of these interventions were effective. Many interventions (*n* = 11, of which 3 effective) had a high intensity, meaning they contained 12 or more hours of vocational rehabilitation. A minority of interventions were solely group interventions (*n* = 3, *n* = 1 effective). Only a few (*n* = 3 of which 1 was effective) contained homework assignments. There were no notable differences in duration, intensity and type of intervention between the effective and the not effective interventions.

**Table 4:** Intervention characteristics and elements

Study	Name of intervention	Sick leave duration before start intervention Mean (SD)	Characteristics • Psychosocial / physical / both • Individual / group / both • Multidisciplinary • Homework	Duration^1^ (D) and Intensity (I)	Aim			Result^2^
					Preparing to RTW • Anamnesis • Formulating rehabilitation plan • Education • Counselling • CBT or ACT • Physical	Finding work • Teaching practical skills • Planning RTW • Placing in work • Helping find work	Remaining in work • Workplace specific exercises • Advising at workplace • Follow-up at workplace	
Berglund et al	Multidisciplinary team	7.7 years	Psychosocial Individual Multidisciplinary No homework	D: Long I: NM	Anamnesis Formulating rehabilitation plan Counselling	NA	NA	+
Blonk et al	Combined intervention	NM	Psychosocial Individual Not multidisciplinary Homework	D: Short I: Moderate	Education Counselling CBT or ACT	Planning RTW	NA	+
Cheng	Workplace-based work hardening training	137.9 days (workdays lost since injury)	Physical Individual Not multidisciplinary No homework	D: Short I: High	Education Physical	NA	Advising at workplace	+
Corey et al	Functional restoration program	4.6 months (duration of disability)	Both Both Multidisciplinary No homework	D: Medium I: High	Formulating rehabilitation plan Education CBT or ACT Physical	NA	NA	+
Della-Posta	CBT	39.4 weeks	Psychosocial Group NM No homework	D: Short I: High	CBT or ACT	NA	NA	+
Drake et al	IPS + systematic medication management + behavioral health services	97.9 months on SSDI	Psychosocial + Medical Individual Multidisciplinary No homework	D: Long I: NM	Anamnesis Counselling	Placed in work	Advising at workplace Follow-up at workplace	+
Fleten et al	Minimal intervention package	231.1 days	Psychosocial Individual Not multidisciplinary No homework	D: NA* I: NA*	Anamnesis Education Counselling	NA	NA	+
Lambeek et al	Integrated care (workplace intervention and graded activity)	NM	Both Both Multidisciplinary No homework	D: Medium I: Moderate	Anamnesis Formulating rehabilitation plan Physical	NA	Advising at workplace	+
Li-Tsang et al	Job placement and support	NM	Psychosocial Both Not multidisciplinary No homework	D: Short I: NM	Anamnesis Formulating rehabilitation plan Counselling	Practical skills Searching for work	Advising at workplace	+
Park et al	Motivational interviewing + functional restoration program = CAU	233.7 (SD 688) days (disability duration)	Both Individual Not multidisciplinary No homework	D: NM I: NM	Formulating rehabilitation plan Counselling	NA	NA	+
Marhold	Cognitive behavioral return-to-work program	Short-term: 3 months Long-term: 26 months	Both Both Not multidisciplinary Homework	D: Medium I: High	Education Counselling ACT or CBT Physical	Placed in work	Physically at workplace Follow-up at workplace	Short-term sick leave (3 months): + Long-term sick leave (26 months): x
Van der Feltz-Cornelis et al	Psychiatric consultation program	144 days	Psychosocial Individual Multidisciplinary No homework	D: NM I: NM	Formulating rehabilitation plan Counselling	Planning RTW	Advising at workplace	3 months: + 6 months: x
Berglund et al	ACT	7.7 years	Psychosocial Individual Not multidisciplinary No homework	D: Unclear I: Moderate	CBT or ACT	NA	NA	x
Blonk et al	CBT	NM	Psychosocial Individual Not multidisciplinary Homework	D: Medium I: Moderate	CBT or ACT	NA	NA	x
Hees et al	Occupational Therapy + TAU	median: 4.8 months	Psychosocial Both Not multidisciplinary No homework	D: Medium I: High	Anamnesis Formulating rehabilitation plan Counselling ACT or CBT	NA	Advising at workplace Follow-up at workplace	6 months: x 12 months: x 18 months: x
Heinrich	Physical Training with a Cognitive Behavioural component and Workplace specific Exercises	8.8 weeks (after 52 weeks waiting time)	Both Group NM No homework	D: Medium I: High	Anamnesis Formulating rehabilitation plan ACT or CBT Physical	NA	Advising at workplace	6 months: x 12 months: x
Hellstrom et al	IPS-MA	NM	Psychosocial Individual Multidisciplinary No homework	D: NM I: NM	Formulating rehabilitation plan Counselling	Practical skills Searching for work	Advising at workplace	12 months: x 24 months: x
Huibers et al	CBT	12.3 years	Psychosocial Individual Not multidisciplinary No homework	D: Medium I: Low	Anamnesis Formulating rehabilitation plan ACT or CBT	Planning RTW	NA	4 months: x 8 months; x 12 months: x
Lytsy et al	Multidisciplinary assessments and individual rehabilitation	7.5 years (SD 3.2)	Both Individual Multidisciplinary No homework	D: Long I: High	Anamnesis Formulating rehabilitation plan Counselling ACT or CBT	NA	NA	x
	ACT	7.5 years (SD 3.2)	Psychosocial Individual Not multidisciplinary No homework	D: Long I: High	ACT or CBT	NA	NA	x
Magnussen	brief vocational-oriented intervention	10.69 years	Psychosocial Both Multidisciplinary No homework	D: Short I: Moderate	Anamnesis Counselling	NA	NA	x
Myhre	Work-focussed intervention + CAU	median 112 days	Psychosocial Individual Multidisciplinary No homework	D: Short I: High	Anamnesis Formulating rehabilitation plan Education	NA	Advising at workplace	x
Nilsson	Occupational therapy and social counselling	median 290 days	Both NM Multidisciplinary No homework	D: Medium I: High	Anamnesis Formulating rehabilitation plan Counselling Physical	NA	Advising at workplace	x
van Egmond	Tailored RTW program	NM	Both Individual Multidisciplinary No homework	D: Long I: Moderate	Anamnesis Formulating rehabilitation plan Counselling Physical	Placed in work	Advising at workplace	x
Heinrich	Physical training	8.8 weeks (after 52 weeks waiting time)	Physical Group Not multidisciplinary No homework	D: Medium I: High	Anamnesis Formulating rehabilitation plan Physical	NA	NA	6 months: - 12 months: x

^1^Duration: short: ≤ 1 month, medium: > 1 month ≤ 6 months, long: > 6 months

^2^Results: +  = significant difference in favour of the intervention group,—= significant difference in favour of the control group; x = no significant effect between groups

*Intervention existed out of a single information package send to the client 14 days after the start of the current sick leave

*ACT* acceptance and commitment therapy, *CAU* care as usual, *CBT* cognitive behavioural therapy, *D* duration, *I* intensity, *IPS* individual placement and support, *NA* not applicable, *NM* not mentioned, *OP* occupational physician, *RTW* return to work, *SD* standard deviation, *SPES* swedish public employment service, *SSIA* swedish social insurance agency, *SSDI* social security disability insurance, *TAU* treatment as usual

All included interventions were aimed at preparing the worker to return to work. Nearly half of the interventions were also aimed at helping the worker find work (8 interventions) or remain in work (12 interventions). Six interventions included all three aims: preparing the worker to RTW, helping the worker find work and helping the worker remain in work. Of the six interventions that included all three aims, two were effective on RTW [42, 45], while the other four were not [25, 33, 40, 44]. In the next paragraphs, the content of the interventions for each main aim is further described.

#### Main Aim 1: Preparing the Worker for Return to Work

Ten of the 25 interventions aimed at preparing the worker to return to work were effective on RTW compared to the usual care. The effective interventions all contained various types of activities that were aimed at preparing the worker to return to work: these often contained a form of counselling, coaching or motivational interviewing (*n* = 6) or education (*n* = 4), and often started with assessing the underlying problems for RTW of the worker (*n* = 5). In general, studies that were aimed at preparing the worker for returning to work often included assessing the underlying problems for RTW of the worker (*n* = 14) and formulating a rehabilitation plan aimed at RTW, which is often based on this assessment (*n* = 11). This, however, was not necessarily an element in effective interventions. Only two interventions that were effective included both of these activities, while ten ineffective interventions included this combination of activities. The interventions that contained a psychological intervention based on CBT, ACT or cognitive restructuring (*n* = 11) were often aimed at managing pain or being able to cope with problems. Only three of these interventions were effective. There was no clear difference in the content of the psychological interventions between effective and not effective studies. The effective interventions that contained a physical aspect included (among others) graded activity [37, 41] and work hardening [27], but again, there was no clear difference in the content of effective and non-effective physical interventions.

#### Main Aim 2: Helping the Worker Find Work

Eight interventions included elements focused on helping the worker find work. Three of these interventions were effective to improve the RTW outcome compared to the usual care (the combined intervention of study [34] and studies [42, 45]. The effective interventions contained planning returning to work with the worker [34], placing the worker in work [45] or teaching practical skills and helping the worker look for work [42]. However, these elements were comparable to those of the ineffective interventions.

#### Main Aim 3: Helping the Worker Remain in Work

Twelve of the interventions included elements focused on helping the worker remain in the workplace, of which four were effective on the RTW outcome compared to the usual care [27, 41, 42, 45]. These effective interventions existed of advising at the workplace [27, 41, 42] or advising on working circumstances at the workplace and a follow-up at the workplace [45]. Other interventions that included advising at the workplace, however, were not effective (e.g. [32, 38]). Both interventions that included physical training at the workplace were not more effective than the usual care ([30] or workplace specific exercises [25]). Also, interventions including a follow-up (booster sessions or ongoing support if needed) at the workplace (e.g. [25]) were not effective. The interventions that were only effective on short-term RTW included physical therapy and following up at the workplace or advising at the workplace and following up at the workplace.

## Discussion

We included 25 interventions that were tested in 21 studies. Ten interventions showed a positive effect on RTW compared to usual care, two interventions showed mixed results, one study showed an effect in favor of the usual care group at 6 months and no effect at 12 months. Twelve interventions were not effective. As for the main aims of the intervention, all effective interventions were aimed at helping the worker prepare to RTW, while one intervention had the additional aim to help the worker find work and two interventions were also aimed at helping the worker remain in work. Two interventions contained all three main aims. The elements analyzed in this study could not explain why some interventions were effective and others were not.

### Comparison with Other Studies

The results show that VR interventions are sometimes more effective and often just as effective than usual care in helping people who have been on long-term sick leave (> 90 days) return to work. This review thus shows that effective interventions also exist for people on long-term sick leave. This is in agreement with earlier reviews, which showed that it is possible to influence return to work of people on long-term sick leave [8, 13]. Earlier reviews [7, 8] also suggested that interventions offered to this group should be high intensive interventions. The identified interventions in our study were often high intensive in terms of contact hours and duration, and most were comprehensive by including multiple aims. However, we could not explain differences in effectiveness by differences in intensity, comprehensiveness or the multiple disciplines included. Additionally, the identified characteristics of the studies, such as duration of the intervention or who provided the intervention, could also not explain the differences in effectiveness of the interventions.

The elements that we found, are comparable to the elements that vocational rehabilitation professionals mentioned as crucial for VR interventions [46]. Especially formulating a rehabilitation plan was often part of the effective interventions (5 interventions). But it was also an element in many not effective interventions (9 interventions). Another review [1] concluded that contact with the workplace and including multiple components, are elements of effective interventions. Our results show that these elements are indeed part of the effective interventions, but were often also a part of the not effective interventions. A qualitative review by Reed et al. [47] studied elements that help ensure that workers experience VR interventions as supportive and effective. These elements are also often included in the effective interventions identified in this review. Especially personalized services (*n* = 4) and collaboration with the employing organization (*n* = 4) are often included. However, Reed et al. also concluded that skills development, and sustainable and ongoing interventions is what workers find supportive and effective, while these elements were only scarcely part of the effective (and the not-effective) interventions included in this review. The perspective of an effective intervention of a worker might thus be different from the actual effectiveness of an intervention.

With this review we contributed to identifying which elements effective VR interventions entail. We could, however, not conclude which elements make a VR intervention effective (or ineffective) in helping people with a long-term sick leave return to work. This study shows that including multiple phases of helping a worker return to work does not mean the intervention will be effective. It is possible that other elements that were not identified in this review do make an intervention effective. This could be due to the often minimal description of the interventions, which made it difficult to determine the elements. Often studies did not report on personal elements, such as motivation, illness perception and societal participation that do have a large influence on whether or not a person on (long-term) sick leave benefits from interventions and returns to work [48]. Studies also often did not report on the context, such as the organization, in which the VR intervention was tested, while this can be of influence on the effectiveness of the intervention [49]. It is also a possibility that the usual care offered in the studies with the not-effective studies were more elaborate than the usual care offered in the studies with the effective VR interventions. Due to the limited description of the usual care we could not determine if this was the case.

### Strengths and Limitations

The main strength of this systematic review is that it is specifically aimed at people with a longer duration of sick leave (> 90 days). It is often thought that VR interventions are not effective or less effective for people with a sick leave duration of more than 90 days. Another strength of this review is that we included populations with any disease type. This is in line with the assumptions of the ICF-model [50] and Gragnano [51], that factors that influence RTW are often the same for people with different diseases. A methodological strength of this study is that we conducted a very broad literature search which limited the chance that we missed important studies, by including a large variety of search terms related to “rehabilitation” and by searching in multiple databases. A limitation of this review is that the included studies often minimally described the intervention and the usual care and only few used a protocol, such as the TIDieR checklist [52], to describe the intervention. Because of this, we were often not able to retrieve all elements in detail and may have missed elements that were relevant, but not described. The included studies were also not always of a high quality, in fact 8 studies had a low quality, often due to a selection bias or weak data collection methods. Another limitation of this study is that only four of the 21 included articles obtained a high (strong) score in the quality assessment. This was mainly due to selection bias, participants that were not blinded for the condition they were allocated to and using self-reported questionnaires to measure return to work.

### Implications for Policy, Practice and Research

Based on the results of this review we first can conclude that people on long-term sick leave can return to work and that VR interventions can contribute to this. With this information, social security institutes and other organizations aimed at vocational rehabilitation have an evidence-base to offer VR interventions to people who have been on long-term sick leave or who receive a disability pension. Based on this review we cannot give an overview of the elements of (effective) VR interventions. Future studies are needed to gain (more) knowledge about what elements make VR interventions effective in different settings for people with long-term sick leave. Only after research has shed more light on this, organizations which offer RTW support can focus on individual elements of interventions. This knowledge can be used in tools to provide VR professionals with evidence-based knowledge on effective VR interventions for people on long-term sick leave to ensure workers receive good VR. Our previous studies [48, 53] showed that a decision aid to identify barriers for RTW and to choose appropriate VR interventions can help in reducing practice variation among VR professionals. This review can improve the decision aid with evidence-based knowledge. Future research should investigate how this information can best be implemented in the decision aid. It is recommended that future studies improve the participation rate and use more reliable methods to measure the main outcome to improve the quality of these studies.

## Conclusions

This study showed that VR interventions can contribute to the RTW of people with a long-term sick leave. But no specific characteristics or elements were found that could explain why an interventions was more effective on RTW than the offered usual care. There is still much to gain in understanding which characteristics or elements of VR interventions that are aimed at helping people with a long-term sick leavereturn to work are effective or not. More knowledge is needed on which elements constitute effective VR interventions for this group tailored to personal and contextual characteristics to facilitate a better perspective on RTW. In order to improve vocational rehabilitation for people on long-term sick leave or receiving a work disability pension, more high quality studies are needed, in which the study population and intervention content is better described in order to be able to identify what elements make the intervention effective for whom.

## Supplementary Information

Below is the link to the electronic supplementary material.

Supplementary file1 (PDF 141 KB)

## Data Availability

The datasets generated and/or analyzed during the current study are not publicly available due to privacy reasons (given the relatively small population of professionals in this field), but are available from the corresponding author (MAH) on reasonable request.

## Abbreviations

VR: Vocational rehabilitation
RTW: Return to work
RCT: Randomized controlled trial
CBT: Cognitive behavioral therapy
ACT: Accteptance and commitment therapy
