# Supplementary material for: Elements of Return-to-Work Interventions for Workers on Long-Term Sick Leave: A Systematic Literature Review
Source: J Occup Rehabil. 2024 Jun 7;35(2):159–80. doi: 10.1007/s10926-024-10203-0 (PMC12089148; doi:10.1007/s10926-024-10203-0)
Supplement: Supplementary file 1 — Supplementary file1 (PDF 141 KB) [file 10926_2024_10203_MOESM1_ESM.pdf]

Elements of return-to-work interventions for workers on long-term sick leave: a systematic literature review

Christa J.C. de Geus, Maaike A. Huysmans, H. Jolanda van Rijssen, Marianne de Maaker-de Berkhof, Linda J. Schoonmade, Johannes R. Anema

Appendix 1: search strategy

Search History PubMed February 7, 2022

Search	Query	Items found
#5	(#1 OR #2) AND #3 AND #4	4,230
#4	"Clinical Trials as Topic"[Mesh] OR "Controlled Clinical Trials as Topic"[Mesh] OR "Controlled Clinical Trial" [Publication Type] OR "Random Allocation"[Mesh] OR ((random*[tiab] AND (controlled[tiab] OR control[tiab] OR versus[tiab] OR vs[tiab] OR group[tiab] OR groups[tiab] OR comparison[tiab] OR compared[tiab] OR arm[tiab] OR arms[tiab] OR crossover[tiab] OR cross-over[tiab]) AND (trial[tiab] OR study[tiab])))	1,372,398
#3	"Rehabilitation, Vocational"[Mesh] OR "Case Management"[Mesh] OR "Occupational Therapy"[Mesh] OR "Counseling"[Mesh] OR "Rehabilitation"[Mesh] OR intervention*[tiab] OR program*[tiab] OR "case manag*"[tiab] OR casemanag*[tiab] OR "occupational therap*"[tiab] OR counseling[tiab] OR counselling[tiab] OR rehabilitation[tiab] OR "absence management"[tiab] OR project*[tiab] OR plan[tiab] OR model[tiab]	4,932,675
#2	"Insurance, Disability"[Mesh] OR ((beneficiar*[tiab] OR pension*[tiab] OR claim*[tiab] OR compensation*[tiab] OR insurance*[tiab] OR security[tiab]) AND ("Disabled Persons"[Mesh] OR sickness[tiab] OR disabilit*[tiab] OR disabled[tiab] OR invalidit*[tiab] OR absente*[tiab] OR absence[tiab] OR illness[tiab]))	34,761
#1	"Absenteeism"[Mesh] OR "Sick Leave"[Mesh] OR "Return to Work"[Mesh] OR "rehabilitation, vocational"[Mesh] OR "Return to Work"[tiab] OR "work resumption"[tiab] OR "back to work"[tiab] OR "work disab*"[tiab] OR "work incapacit*"[tiab] OR "work incapabilit*"[tiab] OR "work capacit*"[tiab] OR "work capabilit*"[tiab] OR "work inhibition*"[tiab] OR "working incapacit*"[tiab] OR "working capacit*"[tiab] OR "medical leave*"[tiab] OR "sick leave*"[tiab] OR "disability leave*"[tiab] OR "absente*"[tiab] OR "work absen*"[tiab] OR "sickness absen*"[tiab] OR "disability absen*"[tiab] OR "sick day*"[tiab] OR "illness day*"[tiab] OR "sick listed"[tiab] OR "reintegration"[tiab] OR "reemployment"[tiab] OR "re-employment"[tiab] OR "job reentry"[tiab] OR "job re-entry"[tiab]	54,084

Search History Embase.com February 7, 2022

Search	Query	Items found
#6	#5 NOT 'conference abstract'/it	3,940
#5	(#1 OR #2) AND #3 AND #4	4,901
#4	'clinical trial (topic)'/exp OR 'controlled clinical trial (topic)'/exp OR 'randomization'/exp OR ((random*:ab,ti,kw AND (controlled:ab,ti,kw OR control:ab,ti,kw OR versus:ab,ti,kw OR vs:ab,ti,kw OR group:ab,ti,kw OR groups:ab,ti,kw OR comparison:ab,ti,kw OR compared:ab,ti,kw OR arm:ab,ti,kw OR arms:ab,ti,kw OR crossover:ab,ti,kw OR ‘cross-over’:ab,ti,kw) AND (trial:ab,ti,kw OR study:ab,ti,kw)))	1,435,337
#3	'vocational rehabilitation'/exp OR ‘case management'/exp OR 'occupational therapy'/exp OR 'rehabilitation'/exp OR 'intervention study'/exp OR 'counseling'/exp OR intervention*:ab,ti,kw OR program*:ab,ti,kw OR ‘case manag*’:ab,ti,kw OR casemanag*:ab,ti,kw OR ‘occupational therap*’:ab,ti,kw OR counseling:ab,ti,kw OR counselling:ab,ti,kw OR rehabilitation:ab,ti,kw OR ‘absence management’:ab,ti,kw OR project*:ab,ti,kw OR plan:ab,ti,kw OR model:ab,ti,kw	6,485,130
#2	('insurance'/exp OR beneficiar*:ab,ti,kw OR pension*:ab,ti,kw OR claim*:ab,ti,kw OR compensation*:ab,ti,kw OR insurance*:ab,ti,kw OR security:ab,ti,kw) AND (‘disabled person'/exp OR sickness:ab,ti,kw OR disabilit*:ab,ti,kw OR disabled:ab,ti,kw OR invalidit*:ab,ti,kw OR absente*:ab,ti,kw OR absence:ab,ti,kw OR illness:ab,ti,kw)	47,626
#1	'absenteeism'/exp OR 'medical leave'/exp OR 'return to work'/exp OR 'vocational rehabilitation'/exp OR 'work resumption'/exp OR ‘return to work’:ab,ti,kw OR ‘back to work’:ab,ti,kw OR ‘work disab*’:ab,ti,kw OR ‘work incapacit*’:ab,ti,kw OR ‘work incapabilit*’:ab,ti,kw OR ‘work capacit*’:ab,ti,kw OR ‘work capabilit*’:ab,ti,kw OR ‘working incapacit*’:ab,ti,kw OR ‘working capacit*’:ab,ti,kw OR ‘medical leave*’:ab,ti,kw OR ‘sick leave*’:ab,ti,kw OR ‘disability leave*’:ab,ti,kw OR absente*:ab,ti,kw OR ‘work absen*’:ab,ti,kw OR ‘sickness absen*’:ab,ti,kw OR ‘sick day*’:ab,ti,kw OR ‘illness day*’:ab,ti,kw OR ‘sick listed’:ab,ti,kw OR reintegration:ab,ti,kw OR reemployment:ab,ti,kw OR ‘re-employment’:ab,ti,kw OR ‘job reentry’:ab,ti,kw OR ‘job re-entry’:ab,ti,kw	72,880

Search History APA PsycInfo (Ebsco) February 7, 2022

Search	Query	Items found
S5	(S1 OR S2) AND S3 AND S4	975
S4	(ZC "clinical trial") OR ((DE ("Clinical Trials" OR "Random Sampling") OR (TI (random*) OR AB (random*) OR KW (random*)) AND (TI (controlled OR control OR versus OR vs OR group OR groups OR comparison OR compared OR arm OR	152,384

Search	Query	Items found
	arms OR crossover OR “cross-over”) OR AB (controlled OR control OR versus OR vs OR group OR groups OR comparison OR compared OR arm OR arms OR crossover OR “cross-over”) OR KW (controlled OR control OR versus OR vs OR group OR groups OR comparison OR compared OR arm OR arms OR crossover OR “cross-over”)) AND (TI (trial OR study) OR AB (trial OR study) OR KW (trial OR study)))	
S3	DE ("Vocational Rehabilitation" OR "Supported Employment" OR "Vocational Evaluation" OR "Work Adjustment Training" OR "Rehabilitation Counseling" OR "Occupational Guidance" OR "Employee Assistance Programs" OR "Counseling" OR "Case Management" OR “Occupational Therapy" OR "Rehabilitation" OR "Counseling" OR "Intervention" OR "Workplace Intervention") OR TI (intervention* OR program* OR “case manag*” OR casemanag* OR “occupational therap*” OR counseling OR counselling OR rehabilitation OR absence management OR project OR plan OR model) OR AB (intervention* OR program* OR “case manag*” OR casemanag* OR “occupational therap*” OR counseling OR counselling OR rehabilitation OR absence management OR project OR plan OR model) OR KW (intervention* OR program* OR “case manag*” OR casemanag* OR “occupational therap*” OR counseling OR counselling OR rehabilitation OR absence management OR project OR plan OR model)	1,577,072
S2	(DE ("Social Security" OR "Insurance" OR "Employee Leave Benefits") OR TI (beneficiar* OR pension* OR claim* OR compensation* OR insurance* OR security) OR AB (beneficiar* OR pension* OR claim* OR compensation* OR insurance* OR security) OR KW (beneficiar* OR pension* OR claim* OR compensation* OR insurance* OR security)) AND (DE ("Disabled Personnel" OR "Disabilities") OR TI (sickness OR disabilit* OR disabled OR invalidit* OR absente* OR absence OR illness) OR AB (sickness OR disabilit* OR disabled OR invalidit* OR absente* OR absence OR illness) OR KW (sickness OR disabilit* OR disabled OR invalidit* OR absente* OR absence OR illness))	13,537
S1	DE ("Employee Absenteeism" OR "Reemployment" OR "Vocational Rehabilitation") OR TI (“return to work” OR “back to work” OR “work disab*” OR “work incapacit*” OR “work incapabilit*” OR “work capacit*” OR “work capabilit*” OR “working incapacit*” OR “working capacit*” OR “medical leave*” OR “sick leave*” OR “disability leave*” OR absente* OR “work absen*” OR “sickness absen*” OR “sick day*” OR “illness day*” OR “sick listed” OR reintegration OR reemployment OR “re-employment” OR “job reentry” OR “job re-entry”) OR AB (“return to work” OR “back to work” OR “work disab*” OR “work incapacit*” OR “work incapabilit*” OR “work capacit*” OR “work capabilit*” OR “working incapacit*” OR “working capacit*” OR “medical leave*” OR “sick leave*” OR “disability leave*” OR absente* OR “work absen*” OR “sickness absen*” OR “sick day*” OR “illness day*” OR “sick listed” OR	21,656

Search	Query	Items found
	reintegration OR reemployment OR “re-employment” OR “job reentry” OR “job re-entry”)OR KW (“return to work” OR “back to work” OR “work* disab*” OR “work* incapacit*” OR “work* incapabilit*” OR “work* capacit*” OR “work* capabilit*” OR “medical leave*” OR “sick leave*” OR “disability leave*” OR absente* OR “work absen*” OR “sickness absen*” OR “sick day*” OR “illness day*” OR “sick listed” OR reintegration OR reemployment OR “re-employment” OR “job reentry” OR “job re-entry”)	

Search History Cinahl (Ebsco) February 7, 2022

Search	Query	Items found
S5	(S1 OR S2) AND S3 AND S4	2,211
S4	(MH "Clinical Trials+" OR "Random Sample+") OR ((TI (random*) OR AB (random*)) AND (TI (controlled OR control OR versus OR vs OR group OR groups OR comparison OR compared OR arm OR arms OR crossover OR “cross-over”) OR AB (controlled OR control OR versus OR vs OR group OR groups OR comparison OR compared OR arm OR arms OR crossover OR “cross-over”)) AND (TI (trial OR study) OR AB (trial OR study)))	486,295
S3	MH ("Rehabilitation, Vocational+" OR "Rehabilitation+" OR "Case Management" OR "Occupational Therapy+" OR "Counseling+") OR TI (intervention* OR program* OR “case manag*” OR casemanag* OR “occupational therap*” OR counseling OR counselling OR rehabilitation OR absence management OR project OR plan OR model) OR AB (intervention* OR program* OR “case manag*” OR casemanag* OR “occupational therap*” OR counseling OR counselling OR rehabilitation OR absence management OR project OR plan OR model)	1,668,832
S2	((MH "Insurance+") OR TI (beneficiar* OR pension* OR claim* OR compensation* OR insurance* OR security) OR AB (beneficiar* OR pension* OR claim* OR compensation* OR insurance* OR security)) AND ((MH "Disabled") OR TI (sickness OR disabilit* OR disabled OR invalidit* OR absente* OR absence OR illness) OR AB (sickness OR disabilit* OR disabled OR invalidit* OR absente* OR absence OR illness))	14,894
S1	MH ("Job Re-Entry" OR "Absenteeism" OR "Vocational Guidance" OR "Sick Leave") OR TI (“return to work” OR “back to work” OR “work disab*” OR “work incapacit*” OR “work incapabilit*” OR “work capacit*” OR “work capabilit*” OR “working incapacit*” OR “working capacit*” OR “medical leave*” OR “sick leave*” OR “disability leave*” OR absente* OR “work absen*” OR “sickness absen*” OR “sick day*” OR “illness day*” OR “sick listed” OR reintegration OR reemployment OR “re-employment” OR “job reentry” OR “job re-entry”) OR AB (“return to work” OR “back to work” OR “work disab*” OR “work incapacit*”	25,734

Search	Query	Items found
	OR “work incapabilit*” OR “work capacit*” OR “work capabilit*” OR “working incapacit*” OR “working capacit*” OR “medical leave*” OR “sick leave*” OR “disability leave*” OR absente* OR “work absen*” OR “sickness absen*” OR “sick day*” OR “illness day*” OR “sick listed” OR reintegration OR reemployment OR “re-employment” OR “job reentry” OR “job re-entry”)	

Search History Scopus February 7, 2022

Search	Query	Items found
#5	(#1 OR #2) AND #3 AND #4	7,498
#4	TITLE-ABS-KEY((random* AND (controlled OR control OR versus OR vs OR group OR groups OR comparison OR compared OR arm OR arms OR crossover OR “cross-over”) AND (trial OR study)))	1,594,275
#3	TITLE-ABS-KEY(intervention* OR program* OR “case manag*” OR casemanag* OR “occupational therap*” OR counseling OR counselling OR rehabilitation OR “absence management” OR project OR plan OR model)	19,494,564
#2	TITLE-ABS-KEY(beneficiar* OR pension* OR claim* OR compensation* OR insurance* OR security) AND (sickness OR disabilit* OR disabled OR invalidit* OR absente* OR absence OR illness)	135,717
#1	TITLE-ABS-KEY(“return to work” OR “back to work” OR “work* disab*” OR “work* incapacit*” OR “work* incapabilit*” OR “work* capacit*” OR “work* capabilit*” OR “medical leave*” OR “sick leave*” OR “disability leave*” OR absente* OR “work absen*” OR “sickness absen*” OR “sick day*” OR “illness day*” OR “sick listed” OR reintegration OR reemployment OR “re-employment” OR “job reentry” OR “job re-entry”)	85,780

Search History the Cochrane Library February 7, 2022

Search	Query	Items found
#5	(#1 OR #2) AND #3 AND #4	4886
#4	(random* AND (controlled OR control OR versus OR vs OR group OR groups OR comparison OR compared OR arm OR arms OR crossover OR “cross-over”) AND (trial OR study)):ti,ab,kw (Word variations have been searched)	1,049,038
#3	(intervention* OR program* OR case NEXT manag* OR casemanag* OR occupational NEXT therap* OR counseling OR counselling OR rehabilitation OR “absence management” OR project OR plan OR model):ti,ab,kw (Word variations have been searched)	689,466

Search	Query	Items found
#2	((beneficiar* OR pension* OR claim* OR compensation* OR insurance* OR security) AND (sickness OR disabilit* OR disabled OR invalidit* OR absente* OR absence OR illness)):ti,ab,kw (Word variations have been searched)	3356
#1	(“return-to-work” OR “back-to-work” OR work* NEXT disab* OR work* NEXT incapacit* OR work* NEXT incapabilit* OR work* NEXT capacit* OR work* NEXT capabilit* OR medical NEXT leave* OR sick NEXT leave* OR disability NEXT leave* OR absente* OR work NEXT absen* OR sickness NEXT absen* OR sick NEXT day* OR illness NEXT day* OR “sick listed” OR reintegration OR reemployment OR “re-employment” OR “job reentry” OR “job re-entry”):ti,ab,kw (Word variations have been searched)	5873
